# Epstein Barr Virus and *Mycobacterium avium* subsp. *paratuberculosis* peptides are recognized in sera and cerebrospinal fluid of MS patients

**DOI:** 10.1038/srep22401

**Published:** 2016-03-09

**Authors:** Giuseppe Mameli, Eleonora Cocco, Jessica Frau, Maria Giovanna Marrosu, Leonardo Antonio Sechi

**Affiliations:** 1 Dipartimento di Scienze Biomediche, Sezione di Microbiologia e Virologia, Università di Sassari, Viale San Pietro 43b, 07100 Sassari, Italy; 2 Centro Sclerosi Multipla, Dipartimento di Sanità Pubblica Medicina Clinica e Molecolare, Università di Cagliari, Via Is Guadazzonis 2, 09126 Cagliari, Italy; 3 Dipartimento di Scienze Mediche “Mario Aresu”, Università di Cagliari, 09126 Cagliari, Italy

## Abstract

*Mycobacterium avium* subsp. *paratuberculosis* (MAP) and Epstein-Barr virus (EBV) epitopes elicit a consistent humoral response in serum of multiple sclerosis patients, but the cross reactivity against the homologous myelin basic protein (MBP) and human interferon regulatory factor 5 (IRF5) has not been searched within the Cerebral Spinal Fluid (CSF). We evaluated in sera and CSF of patients with MS and with other neurological diseases (OND) the humoral response against EBV/MAP peptides and the IRF5/MBP. Our data showed that EBV and MAP peptides are able to induce a specific humoral immune response in MS patients compared to OND controls both in serum and in CSF. An intrathecal specific synthesis of IgG against MBP and their EBV and MAP homologous as indicated by the antibody index was observed in MS patients. The humoral response against EBV, MAP, MBP and IRF5 was significantly higher in MS patients compared to OND both in serum and in CSF. The higher presence of antibodies against MBP and their MAP and EBV homologous in CSF during relapses suggests a possible role of the pathogens in enhancing inflammation.

Multiple sclerosis (MS) is a chronic inflammatory disease of the central nervous system (CNS) resulting in demyelination and neurodegeneration^1^. The disease develops in genetically predisposed individuals in response to environmental factors, most likely viral and bacterial infections^1^. However, recent studies advocate the possible combined roles of *Mycobacterium avium* subsp. *paratuberculosis* (MAP) and Epstein Barr virus (EBV) in the process of autoimmunity inducing MS pathology^2^,^3^. In fact, it was demonstrated that peptides deriving from these pathogens could be cross-recognized by antibodies (Abs) targeting self-epitopes^2^,^3^. In particular it was detected a strong humoral response against peptides from the latent protein of Epstein Barr Virus (EBNA1_400–413)_, the homologous mycobacterial MAP_0106c_121–132_ and the human Myelin Basic Protein (MBP_85–98)_ in MS patients compared to healthy controls (HCs). Also, an interesting common humoral response was reported against a relevant EBV epitope from EBV lytic protein (BOLF1_305–320_) and two homologous peptides belonging to MAP_4027 protein (MAP_4027_18–32_) and Human Interferon Regulatory Factor 5, IRF5 protein (IRF5_424–434_)^3^. A cross-recognition of the mentioned peptides was also demonstrated. In this work we wanted to explore if we could find the same humoral response in CSF and serum against EBV epitopes deriving from EBV lytic and latent proteins, MAP and human homologous proteins in MS patients and in other neurological disease controls. The humoral response against EBV and MAP in CSF could contribute to understand an immunological dysregulatation in the CSF of MS patients. The presence of intrathecal IgGs, is still considered an evidence for the involvement of infectious agents in MS pathogenesis, although their specificity is largely unknown^4^. This study was carried out on samples from patients with MS, inflammatory neurological disease (IND) or non inflammatory neurological disease (NIND) and patients where a diagnosis was not reached, indicated as undetermined neurological disease (UND),aiming to: a) measure circulating serum and CSF Abs against EBV and MAP peptides and their human homologous; b) quantify and correlate the serum IgG levels to the CSF IgG production; c) investigate the IgG cross reaction against the epitopes investigated. Molecular mimicry between immunodominant epitopes deriving from bacterial and viral persistent antigens may be a decisive factor in directing autoimmunity to self-antigens in MS patients. For this reason it was important to explore if the epitopes from EBV and the other homologous MAP antigens were able to induce a humoral reactivity both in CSF and sera. The results could contribute to the understanding of chronic brain inflammation that contribute to MS pathogenesis.

## Results

### CFS/Serum Albumin ratio and Link index

For all samples, Link index as a generic marker of intrathecal IgG synthesis, CSF/serum albumin ratio (Q Alb) as a marker of BBB integrity and percentage of samples with different type of BBB damage were evaluated and shown in Table 1. No statistically significant damage was observed in the BBB of the MS group compared to the other groups as evidenced by the Link index and the CFS/albumin ratio.

### Elisa

**Figure 1:**
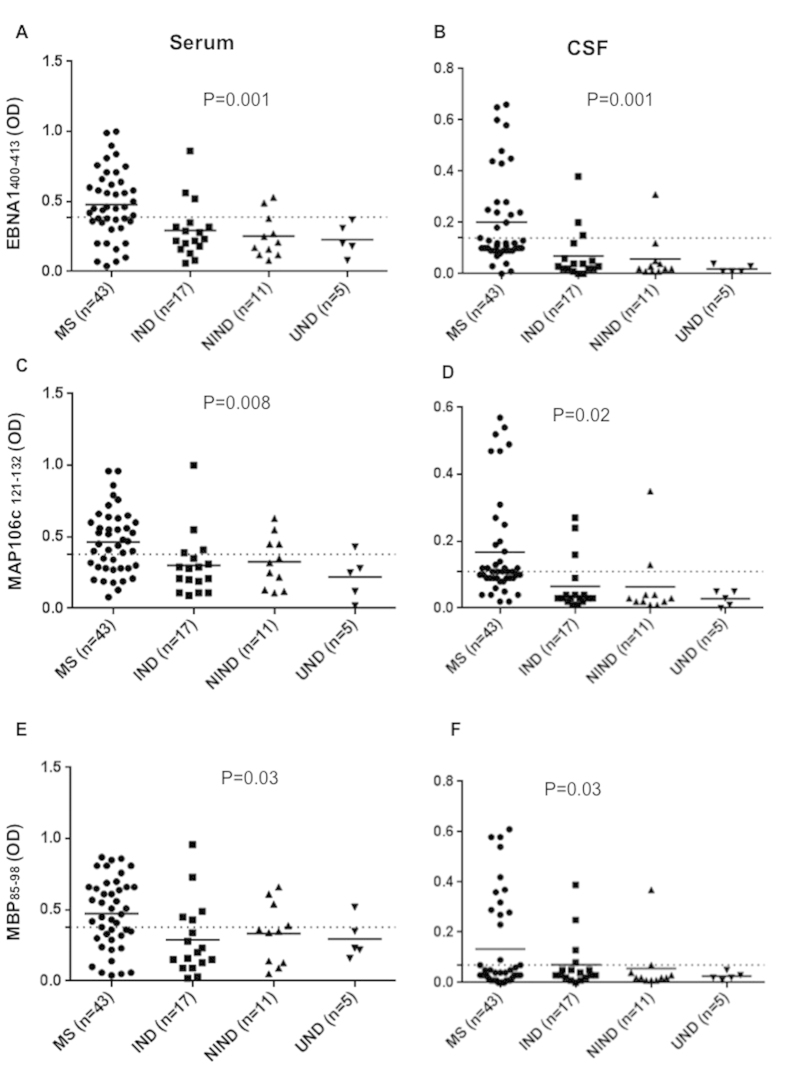
Antibody OD measured by indirect ELISA. Forty-three patients, seventeen IND, eleven NIND and 5 UND were tested for their reactivity against plate-coated with EBNA1_400–413_, MAP_0106c_121–132_, MBP_85–98_, in serum (**A**,**C**,**E**) and in CSF (**B**,**D**,**F**). The horizontal black bars represent the mean value, while *p* values are indicated by two headed arrows drown on the top of each distribution. Cut-off values for positivity, calculated by ROC analysis, are indicated by dashed lines.

Abs against latent and lytic EBV proteins EBNA1 and BOLF1, MAP and Human homologues peptides were monitored in serum and CSF of MS patients and in IND, NIND, UND controls^5^. Abs against EBNA1_400–413_ were found in 26 out 43 (60%) MS patients whereas 3 out of 17 (18%) IND controls, 2 out of 11 (18%) NIND and only one UND were positive in serum (AUC = 0.74, *p* = 0.001) (Fig. 1A). In CSF were found positive 17 out of 43 (40%) MS, 3 out of 33 (10%) IND, none among 11 NIND and 5 UND patients (AUC = 0.80, *p* = 0.001) (Fig. 1B). Regarding MAP_106c_121–132_, 27 out of 43 (63%) MS patients, 4 out of 17 (24%) IND, 4 out of 11 (36%) NIND and one UND were positive (AUC = 0.73, *p* = 0.008) in sera (Fig. 1C), whereas 23 out of 43 (54%) MS patients, 3 out of 17 (18%) IND, 2 out of 11 (18%) NIND and none among UND controls reacted against MAP_106c_121–132_ in CSF (AUC = 0.71, *p* = 0.02) (Fig. 1D). Positivity against MBP_85–98_ was found in 27 out of 43 (63%) MS patients, in 5 out of 17 (29%) IND, 4 out of 11 (36%) NIND and only in one UND in serum (AUC = 0.71, *p* = 0.03) (Fig. 1E). In CSF among the MS subjects, we observed a similar situation where 33% MS, 27% of IND, 10% NIND and 0% of UND patients showed positivity against MBP_85–98_ (AUC = 0.7, *p* = 0.03) (Fig. 1F).

**Figure 2:**
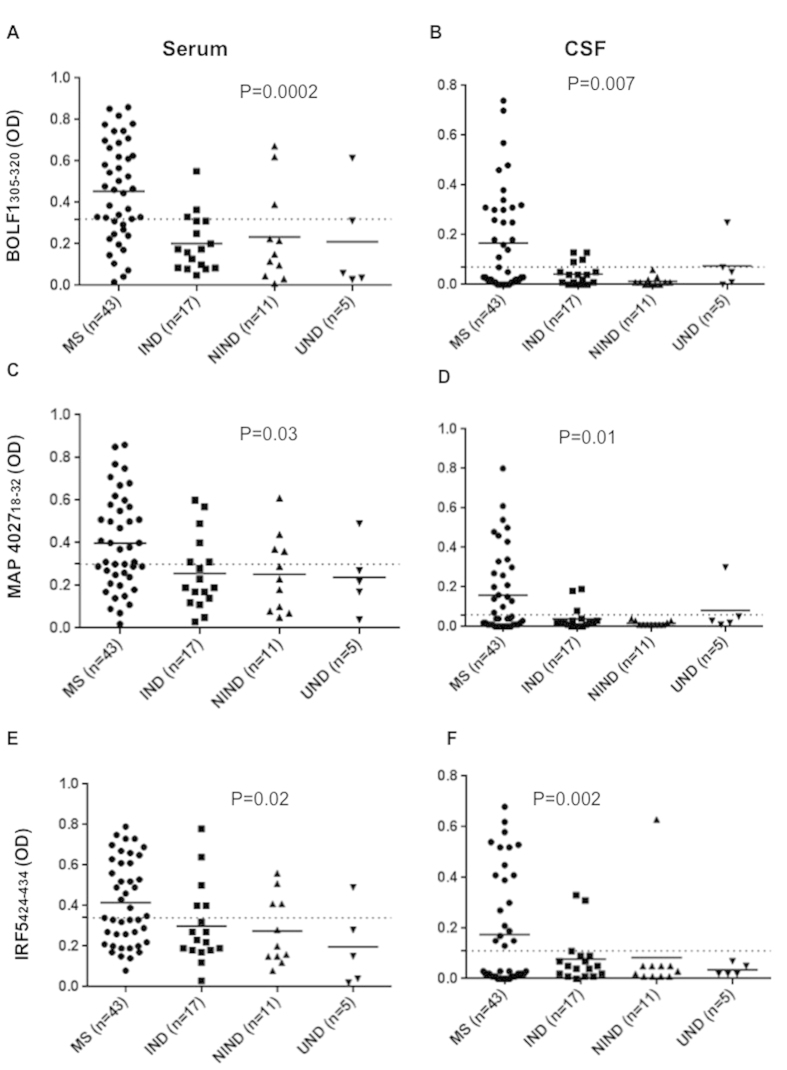
Antibody OD measured by indirect ELISA. Forty-three patients, seventeen IND, eleven NIND and 5 UND were tested for their reactivity against plate-coated with BOLF1_305–320_, MAP_4027_18–32_ and IRF5_424–434_ in serum (**A**,**C**,**E**) and in CSF (**B**,**D**,**F**). The horizontal black bars represent the mean value, while *p* values are indicated by two headed arrows drown on the top of each distribution. Cut-off values for positivity, calculated by ROC analysis, are indicated by dashed lines.

Regarding reactivity against the EBV lytic peptide BOLF1_305–320_ we found 30 out of 43 (69%) MS patients positive, however only 4 out of 17 (23%) IND, 27% NIND and only one UND were positive (AUC = 0.8, *p* = 0.0002) in serum (Fig. 2A); In CSF, 21 out of 43 (48%) MS patients, 4 out of 17 (24%) IND, one UND and none NIND were positive for BOLF1_305–320_ (AUC = 0.65, *p* = 0.007). (Fig. 2B). About the homologous MAP_4027_18–32_, 63% of MS patients, 35% IND, 36% NIND and 20% among the UND controls were positive in serum (AUC = 0.69, *p* = 0.03) whereas in CSF 46% of MS patients, 17% IND, none of NIND and UND controls were positive (AUC = 0.65, *p* = 0.01) (Fig. 2D). Finally, Ab positivity against IRF5_424–434_ was found in 27 out of 43 (63%) MS patients, 5 out of 17 (29%) IND, one NIND (9%) and in one UND control in serum (AUC = 0.67, *p* = 0.02) (Fig. 2F) whereas in CSF 19 out of 43 (44%) MS patients, 2 of 17 (11%) IND, one NIND resulted positive for IRF5_424–434_ (AUC = 0.67, *p* = 0.0002) (Fig. 2F). We considered also a double Abs positivity Serum/CSF in MS samples compared to that found in IND/NIND against the single peptides and their combination (Table 2). A double positivity for EBNA1 and BOLF1 peptides was found among the 33% and 37%, respectively in serum and CSF whereas only 9% of IND/NIND/UND controls was positive (p = 0.025 and 0.007 respectively) (Table 2). Same pattern was observed for the double positivity to MAP_106c_121–132_ and MAP_4027_18–32_ in serum and CSF of MS (37% and 30%, respectively) against the 15% in serum and 4% in CSF of IND/NIND/UND subjects (p = 0.04 and 0.0024 respectively) (Table 2). A strong positive association was found for triple positivity (21%) both in serum and in CSF to BOLF1_305–320_, MAP_4027_18–32_ and IRF5_424–434_ in MS patients in comparison to that (0%) of IND/NIND/UND subjects (p = 0.0042) (Table 2). Moreover, CSF positivity to EBNA1_400–413_, MAP_106c_121–132_ and MBP_85–98_ was associated to MS patients with relapse compared to MS patients without relapse (Table 3).

### Correlation analyses

**Figure 3:**
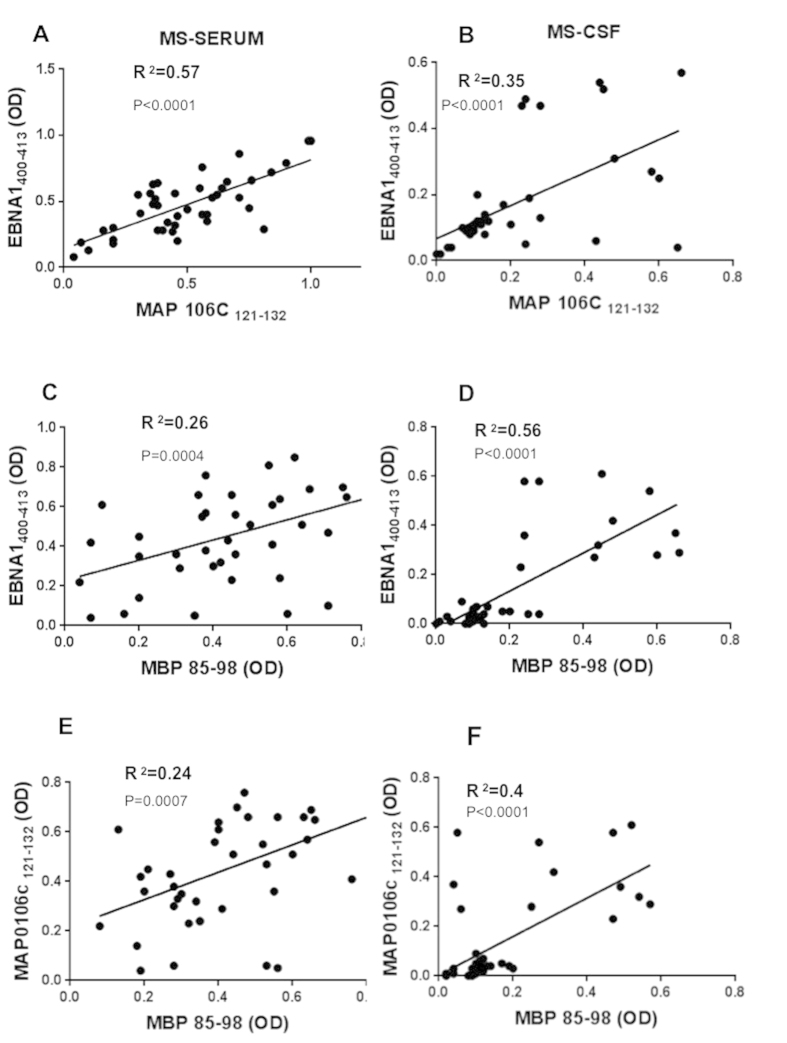
Scatter plot showing the correlations between EBNA1_400–413_ with MAP_0106c_121–132_ in serum (**A**) in CSF (**B**). EBNA1_400–413_ and MAP_0106c_121–132_ with MBP_85–98_ in serum (**C,E**) in CSF (**D,F**) in MS patients. Pearson’s correlation was calculated by Graph Pad 6.

A correlation analyses was performed to establish association between the peptide positivity. We found a good correlation for EBNA1_400–413_ peptide with the homologous MAP_106c_121–132_ peptide in serum (R^2^ = 0.57; P < 0.0001) and a little less (R^2^ = 0.35; p < 0.0001) in CSF (Fig. 3A,B). Interestingly, the correlation analysis showed a stronger correlation between MBP_85–98_ and EBNA1_400–413_ in CSF (R^2^ = 0.56; p < 0.0001) than in serum (R^2^ = 0.26; p = 0.0004) of MS patients (Fig. 2C,D). The same pattern was shown by the correlation between MBP_85–98_ and MAP_121–132_ in CSF (R^2^ = 0.4; p < 0.0001) in comparison to that found in serum (R^2^ = 0.24; p = 0.0007) in MS patients (Fig. 2E,F). These results indicate that the relation of EBNA1_400–413_ and MAP_121–132_ to MBP_85–98_ is stronger in CSF.

**Figure 4:**
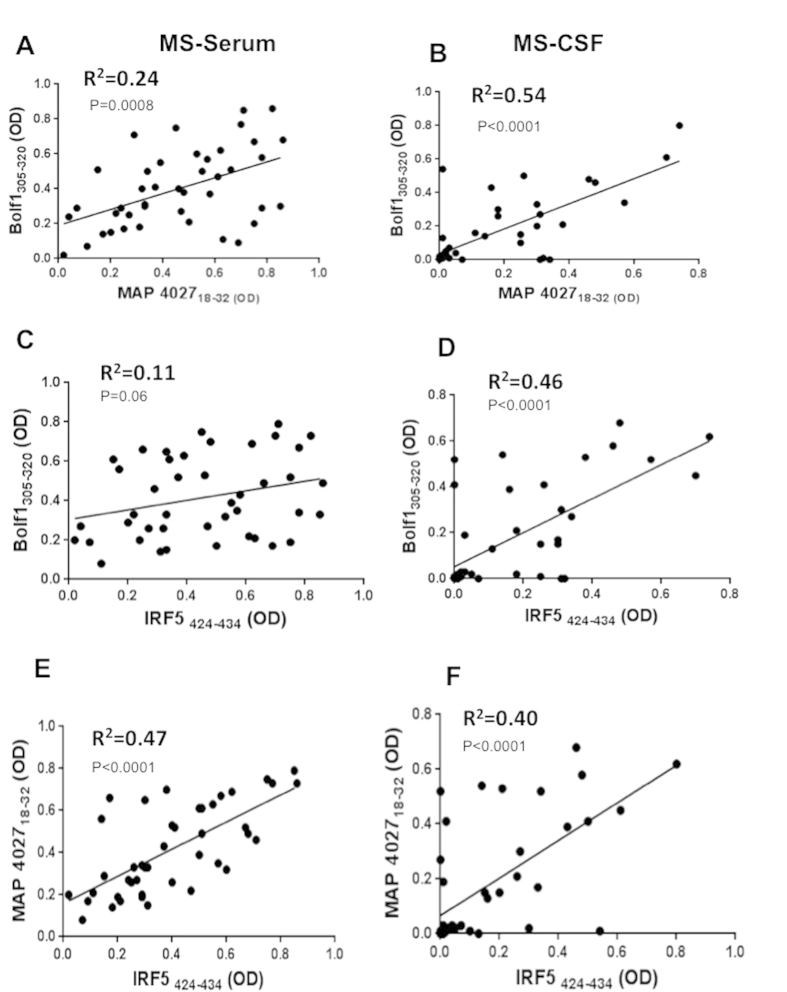
Scatter plot showing the correlations between BOLF1_305–320_ with MAP_4027_18–32_ in serum (**A**) in CSF (**B**). BOLF1_305–320_, MAP_4027_18–32_ with IRF5_424–434_ in serum (**C**,**E**) in CSF (**D**,**F**) in MS patients. Pearson’s correlation was calculated by Graph Pad 6.

We found also a better correlation between Abs against BOLF1_305–320_ and MAP_4027_18–32_ peptides in CSF (R^2^ = 0.54; p < 0.0001) in comparison with that found in serum (R^2^ = 0.24; p = 0.0008) (Fig. 4A,B). Same trend was found also for the correlation between BOLF1_305–320_ and its human homologue peptide IRF5_424–434_ in serum (R^2^ = 0.11; p = 0.11) and in CSF (R^2^ = 0.46; p < 0.0001) (Fig. 4C,D); However the opposite result was observed when we correlate the Ab positivity to MAP_4027_18–32_ and its human homologue peptide IRF5_424–434_ in serum (R^2^ = 0.47; p < 0.0001) and in CSF (R^2^ = 0.40; p < 0.0001).

### Competition assay

**Figure 5:**
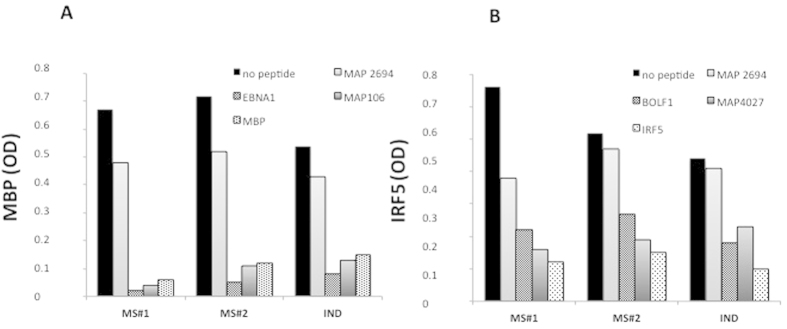
Competition assay with MBP (**A**) and IRF5 (**B**) coated ELISA plates. (**A**) CSF from 2 MS patients and 1 IND were pre-incubated overnight with saturating concentrations [10 μM] of MAP2694_38–46_ (negative control), EBNA1_400–413_, MAP 106_121–132_ and MBP 85–98 (positive control), The first bar represents a regularly performed ELISA (1:2 CSF in PBS-T) peptide. (**B**) The same CSF were pre-incubated with MAP2694_38–46_ (negative control), BOLF1_305–320_, MAP_4027_18–32,_ and IRF5_424–434_ (positive control). The first bar represents a regularly performed ELISA (1:2 CSF in PBS-T) peptide.

To demonstrate the cross-reactivity between MAP/EBV and self epitopes, we developed two competition assays: one coating the MBP_85–98_ on plate and the other coating the IRF5_424–434_ on plate (Fig. 5A,B, respectively). Concerning MBP_85–98_ -coated competition assay (Fig. 5A), in all the CSF subjects (MS#1, MS#2 and IND), both EBNA1 and MAP106 efficiently inhibited antibody binding to the MBP-coated peptide. In IRF5_424–434_-coated competition assay (Fig. 5B), both BOLF1 and MAP 4027 efficiently inhibited IRF5_424–434_-coated binding in all the CSF subjects (MS#1, MS#2 and IND), while the control peptides MAP 2694 did not cause any decrease in signal (Fig. 5A,B). Taken together, these results demonstrate, that Abs anti-EBNA1 and anti-MAP106 target the same conformational MBP epitope and also demonstrate that Abs anti-BOLF1 and anti-MAP 4027 targeting the same conformational IRF5 epitope are cross-reactive in CSF.

### Peptide IgG-specific antibody index (AI) in MS patients

Antibody Index (AI) for all MS patients was calculated with the following formula AI = QIgG[spec]/Qlim^7^. A high number of positive samples against the selected peptides in the MS group had an AI > 1.5 that demonstrate an intratecal IgG-specific antibody production against EBNA1_400–413_, MAP 106, MBP_85–98_ and BOLF1_305–320_, MAP_4027_18–32_, IRF5_424–434_ peptides, as shown in Table 4.

## Discussion

MS aetiology remains unclear, and the current accepted theory for its pathogenesis assigns the main role to the destruction of the myelin-proteolipid shell of axons resulting in autoimmune reactions supposedly induced by a different viral or bacterial infection^1^,^6^,^8^. Although activation of the T-cell response has a crucial role in MS pathogenesis, the B-cell response is equally responsible for the development of the disease by the enhanced synthesis of immunoglobulins usually IgGs^9^. It has been shown that the CSF from MS patients contains oligoclonal immunoglobulins (IgG), which are synthesized within the central nervous system and presumably related to the immune dysfunction, a characteristic feature of MS. The aim of this study was to investigate the presence of a specific humoral response mounted against peptides derived from EBV and MAP antigens homologues to host proteins in serum and in CSF of MS compared to Inflammatory and No inflammatory neurological disease. We investigated different aspects of the association of CSF and serum EBV and MAP positivity in MS and controls. Our studies reported the ability of specific peptides from EBNA1, BOLF1, MAP and their human homologues to induce a strong humoral response in MS patients in comparison to healthy controls, and the existence of a cross-recognition between Abs recognizing these homologues peptides^10^,^11^,^12^. Also these works showed up that EBV and MAP are capable of inducing the production of autoantibodies targeting different MS correlated epitopes^13^. In this study we look for the presence of intrathecal immune response against EBV, MAP and the homologous human peptides. To check the BBB integrity, the Antibody Index (AI) as the marker of a specific intrathecal IgG synthesis against the selected peptides and CSF/serum albumin ratio (Q Alb) were evaluated. Results showed an high number of MS patients with intrathecal IgG synthesis against EBV and MAP with a AI > 1.5, this associated with a better BBB integrity in MS patients in comparison to that of IND, NIND and UND controls (Table 1) support a possible implication of MAP and EBV in MS development. ELISA analysis showed that IgGs against peptides from EBV, MAP and human homologues peptides such as MBP_85–98_ and IRF5_424–434_ were higher in MS patients than in IND/NIND/UND controls in both CSF and serum. Interestingly, a higher correlation was found between the levels of IgGs against EBV, MAP and their homologous peptides in CSF rather than in serum^5^,^10^,^12^. Therefore, we set up a competition assay between the human peptides and their homologous from EBV and MAP in CSF. Our results highlighted that autoantibodies recognizing MBP and IRF5 were able to cross-react with the homologous peptides from EBV (EBNA1, BOLF1) and from MAP (MAP106, MAP4027), possibly by a molecular mimicry mechanism.

The soundness of the results was confirmed by the correlation results, the fact that a triple positivity was found, both in serum and in CSF, against BOLF1_305–320_, MAP_4027_18–32_ and IRF5_424–434_ in MS patients in comparison to the absence in IND/NIND/UND subjects confirmed our hypothesis that MAP and EBV could ignite the autoimmune process in MS and positivity to different peptides (EBNA1_400–413_, MAP_106c_121–132_ and MBP_85–98_) in CSF could be related to MS patients with relapse compared with MS patients without relapse.

Sundström *et al.* also considered as a risk factor the interaction of antibodies against EBNA-1 specific domains and HLA DRB1*1501^14^. In our hands we didn’t find any correlation between a specific HLA and the immunoreactivity with our peptides (data not shown). Recently, another group showed that a productive EBV infection in the peripheral blood could facilitate entry of viral particles and/or newly infected B cells into the CNS^15^. This finding may support our result on the specific ABs against EBV peptides in CSF of MS patients. Our findings emphasize the significance of CSF and serum levels of anti-EBV and MAP IgG. Furthermore, the cross-reaction of MBP_85–98_ supports the possible role of IgG against myelin basic protein in MS disease. Therefore, it is not surprising that several studies report a statistically significant correlation of Abs titers of MBP in MS patients rather than in other neurological disease controls^5^. This data suggest that EBV and MAP may be capable of inducing the production of autoantibodies targeting different MS correlated epitopes expressed in CNS. In conclusion the detection of anti-EBV and anti-MAP IgG in MS patients provided additional evidence of the possible synergistic role of these microrganisms to induce the pathology. Thus, the detection of Abs against EBV and MAP in CSF of MS patients can be considered as an additional criterion (immunological parameter) crucial to better understand MS pathology.

## Materials and Methods

### Subjects

The study protocol was approved by the ethic committee of the University of Cagliari, Italy. All the methods were carried out in “accordance” with the approved guidelines. All the participants provided written consent. CSF and serum samples prospectively collected from MS and Other Neurological Disease composed (OND) by Inflammatory Neurological Diseases (IND), Non Inflammatory Neurological Diseases (NIND) and Undetermined Neurological Diseases (UND) patients were obtained for diagnosis purposes and measured under equal conditions. CSF samples were obtained by a traumatic lumbar puncture performed for diagnosis purposes in the absence of contraindications. All MS samples derived from 43 patients (24 females and 19 males; mean age ± SD was 39 ± 14.0 years) that fulfilled the revised McDonald diagnostic criteria^16^. At the time of the study, 39 patients were diagnosed as relapsing remitting MS, 4 as secondary progressive MS. The duration of the disease was 6 ± 5.4 years, the average age for MS onset was 29.0 ± 10.5 and the expanded disability status scale (EDSS) values ranged from 0 to 7.0 with a 1.9 average. Other neurological diseases (OND) serum and CSF samples were collected from 33 patients (14 females and 19 males) so divided: 17 IND (5 females and 12 males, mean age ± SD was 39 ± 23.1 years), 11 NIND (6 females and 5 males, mean age ± SD was 41 ± 27.1 years) and 5 UND (2 females and 3 males, mean age ± SD was 71 ± 11 years).

### Peptides

Synthetic peptides derived from EBV antigens (BOLF1_305–320_, EBNA1_400–413_), MAP homologues antigens (MAP_4027_18–32_, MAP_0106C_121–132_) and human homologues (MBP_85–98_, IRF5_424–434_) were included in the study^3^,^4^; peptides were synthesized commercially (LifeTein, South Plainfield, NJ 07080 USA) with a purity >90% and kept frozen in single-use aliquots [10 mM] at −80 °C.

### ELISA

Indirect Enzyme-Linked Immunosorbent Assays (ELISA) was carried out to detect specific Abs for all the synthetic peptides (assayed at 10 μg/ml) included in the study as previously reported^17^,^18^ indirect ELISA was set up to detect specific Abs for all peptides. Ninety-six-well Nunc immunoplates were coated overnight at 4 °C with 10 μg/ml of peptides diluted in 0.05 M carbonate–bicarbonate buffer, pH 9.5 (Sigma). Plates were then blocked for 1 h at room temperature with 5% non-fat dried milk (Sigma) and washed twice with phosphate-buffered saline (PBS) containing 0.05% Tween-20 (PBS-T). CSF samples were subsequently added at 1:2 dilution in PBS-T for 2 h at room temperature, while serum were added at 1:100 dilution. After 5 washes in PBS-T, 100 μl of alkaline phosphatase-conjugated goat anti-human immunoglobulin G polyclonal Ab (1:1000; Sigma) was added for 1 h at room temperature. Plates were washed again 5 times in PBS-T and para nitrophenylphosphate (Sigma) added as substrate for alkaline phosphatase. Plates were incubated at 37 °C in the dark for 3–6 min and the absorbance at 405 nm read on a VERSATunable Max microplate reader (Molecular Devices). Negative control wells were obtained by incubation of immobilized peptides with secondary Ab alone, and their mean values subtracted from all samples. Positive control sera were also included in all experiments. Results are expressed as means of triplicate 405 nm optical density (OD) values.

### Competion assay

Two MS and one IND CSF were subjected to ELISA on plates coated with MBP_85–98_ or IRF5_424–434_ respectively. Competitive assays were performed by pre-incubating the CSF overnight at 4 °C with saturating concentrations of peptides [10 μM] (MBP_85–98_; EBNA1_400–413_; MAP 106) and (IRF5_424–434_; BOLF1_305–320_, MAP_4027_18–32_), sero-negative peptide (MAP 2694) was used as negative control [10 μM] and, CSF ELISA for, MBP_85–98_ or IRF5_424–434_ [10 μM] was a positive control.

### IgG -specific antibody index (AI)

Intratecal synthesis of EBNA1_400–413,_ MAP 106, MBP_85–98_ and BOLF1_305–320_, MAP_4027_18–32_ and IRF5_424–434_ IgG-specific antibodies were determined by the antibody index (AI)^7^,^19^,^20^. Data were normalized against a strongly positive control included in all experiments, the reactivity of which was set at 10,000 arbitrary units (AU)/ml. Antibody index by involving albumin concentrations in serum and CSF showed a normal blood/CSF barrier (QIgG[total] > Qlim)^7^. We applied the following formula: AI = QIgG[spec]/Qlim because with QIgG[spec] = IgGspec[CSF]/IgGspec[serum] and Qlim^21^ = 0.93*√(Qalb)^2^ + 6^2^*10^−6^−1.7^2^ *10^−3 ^7,21.

### Statistical analysis

Statistical analysis was performed using GraphPad Prism 6.0 software (San Diego, CA, USA). P-values used to compare different categories in the univariate analysis were calculated by the Mann–Whitney U test. Differences with *p* < 0.05 were considered statistically significant. The diagnostic value of the indirect ELISA assays was evaluated by the receiver operating characteristic (ROC) curve. The optimal cut off values was chosen according to ROC analysis, setting specificity at 90% (i.e., Ab + HCs ≤0.5%) for the MS patients. Pearson’s correlations test among continuous variables were calculated by GraphPad Prism 6.0.

## Additional Information

**How to cite this article**: Mameli, G. *et al.* Epstein Barr Virus and *Mycobacterium avium* subsp. *paratuberculosis* peptides are recognized in sera and cerebrospinal fluid of MS patients. *Sci. Rep.* **6**, 22401; doi: 10.1038/srep22401 (2016).

## Figures and Tables

**Table 1:** Link index as a generic marker of intrathecal IgG synthesis, CSF/serum albumin ratio (Q Alb) as a marker of BBB integrity and percentage of samples with different type of BBB damage are shown.

Samples	Link Index	Q Alb	% BBB damage
MS (43)	**0.45** ± 0.38	**5.41** ± 2.50	70% no 19% low 11% medium
IND (17)	0.28 ± 0.40	6.9 ± 9.5	30% no 23% low 35% medium 12% high
NIND (11)	0.21 ± 0.22	8.5 ± 11.1	82% no 18% low
UND (5)	0.11 ± 0.04	7.8 ± 2.5	40% no 40% low 20% medium

**Table 2:** The table shows MS and IND/NIND/UND samples positive serum/CSF for the peptides and their homologous combination.

Peptide name	MS samples positive serum/CSF %	(IND-NIND-UND) samples positive serum/CSF %	P Value
(EBNA1)	33	9	**0.025**
(BOLF1)	37	9	**0.007**
(MAP 0106)	37	15	**0.04**
(MAP 4027)	30	3	**0.0024**
(MBP)	26	15	0.39 N.S
(IRF5)	30	6	**0.001**
(EBNA1/MBP)	20	9	0.33 *N.S*
(BOLF1/IRF5)	28	3	**0.001**
(MAP 0106/MBP)	19	12	0.53 *N.S*
(MAP 4027/IRF5)	23	3	**0.02**
(EBNA1/MAP 0106/MBP)	14	9	0.7 *N.S*
(BOLF1/MAP 4027/IRF5)	21	0	**0.0042**

P value of MS patients and IND/NIND/UND was calculated by Fisher’s exact test, GraphPad Prism 6.0 software (San Diego, CA, USA).

**Table 3:** The table shows MS with and without relapse samples positive CSF for the EBNA1_400–413_, MAP_0106c_121–132_ and MBP_85–98_ peptides and their combination.

Peptide name	MS with relapse positive CSF %	MS without relapse positive CSF %	P Value
(MBP)	13/23 (57%)	3/20 (15%)	**0.01**
(EBNA1/MBP)	10/23 (43%)	3/20 (15%)	**0.05**
(MAP 0106/MBP)	10/23 (43%)	2/20 (10%)	**0.02**

Fisher’s exact test was calculated by GraphPad Prism 6.0 software (San Diego, CA, USA).

**Table 4:** Antibody Index (AI) as specific marker of intrathecal IgG synthesis for each peptide.

Peptide name	MS samples AI > 1.5%	MS samples positive serum/CSF AI > 1.5
(EBNA1)	33	14/14 (100%)
(BOLF1)	31	13/16 (81%)
(MAP 0106)	33	14/16 (88%)
(MAP 4027)	29	12/13 (92%)
(MBP)	24	10/11 (90%)
(IRF5)	26	11/13 (85%)

The table shows the percentage of all MS patients with AI > 1.5 and the percentage of double positive (in serum and CSF) of MS patients with a AI > 1.5.
